# Supplementary material for: Human confidence judgments reflect reliability-based hierarchical integration of contextual information
Source: Nat Commun. 2019 Nov 28;10:5430. doi: 10.1038/s41467-019-13472-z (PMC6882790; doi:10.1038/s41467-019-13472-z)
Supplement: Supplementary file 1 — Supplementary Information [file 41467_2019_13472_MOESM1_ESM.pdf]

Supplementary information for

‘Human confidence judgments reflect reliability-based hierarchical integration of contextual information’

by Schustek et al.

Supplementary methods

- **Construction of stimuli**

All sample points to be displayed were separated by color and arranged along a horizontal line. The horizontal extent of the grid has a random number of entries, but always more than the maximum number of samples used over the entire session. We randomly sampled two sub-regions along the horizontal direction which are large enough to accommodate both the red and blue sample circles. Within each sub-region, the grid entries are randomly populated by the respective subsample. In the vertical direction, we linearly divided a randomly chosen range by the same amount of grid entries as for the horizontal direction. We then randomly assigned the circles to these positions. The circle density is not preserved over different sample sizes, but roughly for each subsample. Across trials, blue samples are randomly chosen to be either to the left or right side of the red samples.

- **Experiment 1: Instructions**

We emphasized in colloquial terms that the sampling of the passengers is independent and identically distributed (i.i.d.) and that it does not favor either group of passengers. In addition, it was stressed that the sample positions are irrelevant to the task. Our participants were asked to report their decision and their confidence in the correctness of that decision. Specifically, a higher decision confidence should lead to a placement farther from the center whereas for guessing, it should be in the middle. They were specifically advised to rely on their intuition while we discouraged any explicit mental arithmetic. We made it clear that for Experiment 1, there is no relationship between the airplanes (trials).

Regarding the base rates, we mentioned that there are just as many airplanes with a red than with a blue majority arriving at the airport. And that most airplanes are known to have a roughly equal number of passengers of the two kinds on board. Apart from the instructions, the participants could ask any questions to the experimenter they deemed necessary to understand the task.

- **Experiment 2: Instructions**

At the beginning of session 2, each participant read further written instructions which introduced the block-wise design. We explained that there is an event in the city (e.g. a concert, a football match, etc.) that tends to attract e.g. many more red than blue passengers. That the airplanes would be presented one after the other in consecutive trials grouped together in a block separated by pauses. To make that clear, we additionally added visual indication of the in-block trial by presenting five horizontally equidistantly spaced open circles which turned to solid circles one-by-one as the participant progresses through the trials within a block.

We mentioned that the tendency for ’red’ or ’blue’ airplane majorities changes unpredictably from city to city and does not favor either group. Moreover, even though red passengers might preferably travel to a particular city, occasionally there might be airplanes with a blue majority. We attempted to make it very clear that the decision is still about a given airplane majority (trial) and not about the overall tendency of one kind to travel to that city (or airport).

- **Sensory noise**

The task design results in low levels of perceptual noise, which may obscure accurate perception of the sufficient statistics (N_R, N_B). In the basic task (Experiment 1), optimal decisions should always follow the sample majority. However, the misclassification rate, i.e. the proportion of trials where the participant’s binarized response (the decision) was at odds with sample majority were infrequent (mean

3.05% across participants, standard deviation 2.72%; Supplementary Figure 5). If there is sensory noise, the internal estimate would deviate from the sample proportion. In particular, trials whose sample proportion is close to $N_B/N = 0.5$ would lead to suboptimal choices. We assumed normally distributed noise centered on the sample proportion and estimated the standard deviation parameter by fitting a cumulative normal distribution to the psychometric choice curve. The median across participants is 0.034 ,95%-CI (0.025, 0.043), indicating low levels of perceptual noise, so that participants are rarely found to make suboptimal choices.

We assumed two types of noise: a numerosity noise ϵ_s (gaussian noise whose standard deviation scales with sample size $N\sigma_s$)[1], and a sample size - independent noise ϵ_0 (fixed standard deviation noise σ_0). The decision rule with such noise amounts to comparing the difference estimate with 0, where $\delta = N_B - N_R + \epsilon_0 + N\epsilon_s$

The probability of a "blue majority choice" under such rule is:

$$p(blue) = \phi(\frac{N_B - N_R}{\sqrt{\sigma_0^2 + N^2\sigma_s^2}})$$

We fitted parameters $\sigma_0$and σ_s using such decision rule, on concatenated data from all participants to gain statistics (given the low number of misclassified responses). Fitted values indicated fairly low value for both sources of noise: $\hat{\sigma}_0 = 0.72$ and $\hat{\sigma}_s = 0.0115$. As a result, perceptual and decision noise were unlikely to play a major role in behavioral responses on both experiments (including the confidence estimate).

- **Task-intrinsic noise**

To estimate non-input related response noise that is intrinsic to the task, we searched for all trials with the same sufficient statistic. The only assumption we made is symmetry, i.e. decision confidence should only be a function of the absolute distance from the decision boundary (e.g. $q = 0.25$ and $q = 0.75$ result in the same decision confidence). Consequently, with respect to the sample majority, trials of the same sample proportion can be pooled. If there are ten or more trials for a particular sufficient statistic, we computed their squared deviation from the mean. Subsequently, to estimate the variance for fixed inputs, all squared deviations calculated this way were pooled and the mean is taken individually for each participant. The standard deviation parameter of a corresponding Gaussian distribution is estimated by taking the square root. A median value across participants of 0.104 (95 %-CI, (0.086, 0.129)) indicates low to moderate noise levels in Experiment 1.

- **Robust estimation of variation of the response distribution**

For robustness, we estimated a trimmed SD, i.e. we removed values below or above three interquartile ranges from the lower or upper quartile respectively. On the remaining (non-outlying) trials, the ML estimator of the standard deviation for the normal distribution, $\theta = \sqrt{1/N \sum_t (y_t - \hat{y}_t)^2}$, corresponding to the root mean squared deviation (RMSD) of the residual responses is used. A more ideal solution would be to set θ so as to strictly maximize the likelihood of the responses for the truncated Gaussian. For the sake of faster computations however, we resort to this approximate approach which is justified by the relatively low behavioral response noise (see 'Task-intrinsic noise' section above).

- **Cross-validation splits**

As cross validation is a computationally expensive method, we use a random 5-fold split of the data into training and test sets such that each training point is used four times for training and once for testing. However, to avoid splits that are highly unrepresentative of the response distribution, we used a stratified

version of CV to ensure that the mean response $\langle y \rangle$ is approximately equal in all folds. For this purpose, we assigned the data points to one of the q cumulative quantiles of the response distribution. We then constructed slices that contain one value from each cumulative quantile. Subsequently, we sampled the slices to create the 5-fold CV splits. The number of quantiles q is chosen from suitable multiples of the factors of the number of trials close to eight.
To improve the reliability of the per participant estimates of the model evidence (CVLL), we repeated this procedure five times with different random splits and aggregated the output so that in total 25 CV folds are performed for each participant and model. For the prior learning task (Experiment 2), only blocks of trials are split. We basically applied the same logic as before to blocks and attempted to achieve an approximately equal amount of trials from all quantiles of the experimental distribution of the decision confidence $|y_t - 0.5| + 0.5$.

- **<u>Experiment 1: Predictions of heuristics models</u>**

Besides Bayesian model selection techniques, we also assessed whether heuristics models (the ratio model and the difference model) could qualitatively capture the dependence of the psychometric curve on sample size. We used the simulated responses from both models (with fitted distorted sigmoid) and applied the same regression analysis as presented in Figure 2b. On top of the simulation approach, we also derived analytically how the slope should vary with sample size according to both models (see section '*Analytical approximations for Experiment 1*'). By definition, the ratio model was completely agnostic to sample size, and was unable to capture the dependence observed in participants (Supplementary Figure 3a). In the difference model, we established that the slope grows linearly with sample size, while the slope grows sublinearly according to the optimal model (and in participants). This lead to a lower slope for low sample size and larger slope for large sample size than observed in participants. In summary, the models predicted a different exponent in the power law for the relation between sample size and slope: 0 for the ratio model (no dependence on sample size); 1 for the difference model (linear dependence); and 0.5 for the optimal model (sublinear dependence). Extracting the power law for each participant thus provides a single metric for sensitivity to sample size that can characterize whether each participant behaved more like the ratio, difference or optimal model. To test this, we fitted the dependence of the slope on the sample size by a power law for participants and for the output of the optimal and heuristic models (Supplementary Figure 3c). The power law rule took the following form:

$$y = (1 + \exp(-aN^b p_b))^{-1}$$

So the slope of the dependence on the proportion of blue passengers was modelled as a power law of sample size aN^b. The fitting was performed using the same truncated gaussian noise model as throughout the study. Results are presented in Supplementary Figure 3.

- **<u>Experiment 2: Compliance with hierarchical task</u>**

Even though our participants performed the hierarchical task remarkably well, few of them showed such little dependence on previous trials within a block that one may doubt whether they properly understood the hierarchical nature of the task. To evidence this, we fitted a linear function $y = a_q(b)q + a_0(b)$ of the sample proportion q to their responses y conditional on the actual block tendency (see Fig. 5a). The separation of the offset $\Delta a = a_0(b = 1) - a_0(b = 0)$ should be significantly positive. We repeated this fit 10000 times with a randomly shuffled assignment of the b-variable for every participant. To derive the p-value that Δa is significantly larger than chance, we compute the fraction that Δa is larger than the surrogates from the shuffling test (see Supplementary Table 1).

- **Experiment 2: Influence of the previous block**

Our paradigm differs from previous studies where context changed during the course of a session in that subjects were explicitly told that context could change at the beginning of each block. As such, subjects did not have to detect changes of environments (the ‘change detection’ problem) but simply could treat each block independently. As such, their response should be biased only from previous stimuli within the block, but not by previous stimuli from previous blocks. To assess whether subjects actually discarded information from previous block, we run the same logistic regression analysis as in Figure 7, extending the trial lag to 4 in all cases. For the first trial in a block, all of these 4 trials corresponded to trials in the previous block; for the second trial in a block, lags t-4 to t-2 correspond to previous block, lag t-1 to the current block, etc. Results are presented in Supplementary Figure 9.

- **Proper normalization of messages**

Here we will focus on finding the normalization constant ψ of Equation (9). Marginalizing out all random variables (integration over the full range of μ_T) must result in the expression being equal to one. Because of independence, the categorical distribution over N factorizes and separately integrates to one. Compact expressions can be found for the μ_T-terms as the product of the distributions $p(D_T|\mu_T)p(\mu_T|b)$ in the integrand is a product of a Binomial distribution and a Beta-distribution. Hence, the resulting distribution is of Beta-shape again but is not normalized. If we drop the index T, the expression for $b = 1$ can be re-written in terms of the gamma function Γ:

$$p(D|\mu)p(\mu|b=1) = \mu^{N_B+\nu_1-1}(1-\mu)^{N_R+\nu_2-1}$$
$$= \text{Beta}(\mu|N_B+\nu_1, N_R+\nu_2)\frac{\Gamma(N_B+\nu_1)\Gamma(N_R+\nu_2)}{\Gamma(N_B+\nu_1+N_R+\nu_2)} \quad (1)$$

To determine ψ, we enforce the normalization condition $1/\psi \sum_b M(b) \int p(D|\mu)\, p(\mu|b)\, \mathrm{d}\mu = 1$. Together with the probability distribution $M(b)$, which can be easily normalized, we arrive at:

$$\psi = \frac{\Gamma(N_B+\nu_1+N_R+\nu_2)}{M(1)\Gamma(N_B+\nu_1)\Gamma(N_R+\nu_2)+M(0)\Gamma(N_B+\nu_2)\Gamma(N_R+\nu_1)} \quad (1)$$

The messages to update the belief $M(b)$ about the block tendency Equation (7) can be normalized analogously.

- **Analytical approximations: general approach**

In order to allow comparisons between the qualitative features of the different models for both experiments, we want to characterize these models in terms of two reduced features:

- The sensitivity of the model response to the proportion or blue passengers (assessed in participants as the slope of psychometric function). This dependence can be modulated by sample size
- A certain bias to respond to either side, capturing in Experiment 2 how previous trials from the same block, grouped in the top-down message *M(b)*, can shift the response for the current trial. Such bias will therefore mainly depend on previous stimuli.

We will look for approximations of all algorithms in the form of sigmoids $c(B) = \sigma(\kappa(p_B - .5 + \beta))$,

where σ is the logistic sigmoid, i.e. $\sigma(x) = (1 + e^{-x})^{-1}$, p_B is the proportion of blue dots in the current sample. In this format, κ is the slope or sensitivity, and β is a bias towards one side.

- **Analytical approximation for Experiment 1**

Optimal inference with beta prior

$$c(B) = \int_{\mu>0.5} Beta(\mu;\ \nu + N_B, \nu + N_R) d\mu$$

It is a regularized incomplete beta distribution. Using a gaussian approximation to the beta distribution:

$$c(B) \approx \int_{\mu>0.5} N\left(\mu;\ \frac{\nu + N_R}{2\nu + N}, \frac{(\nu + N_B)(\nu + N_R)}{(2\nu + N)^2(2\nu + N + 1)}\right) d\mu$$

Using $\int_{-\infty}^{a} N(x; m, v)dx = \ \phi(\frac{a-m}{\sqrt{v}})$, where φ is the cumulative normal distribution, and since $0.5(2\nu + N)$- $\nu + N_R$= $0.5(N_B - N_R)$

$$c(B) \approx \phi((N_B - N_R)\sqrt{\frac{2\nu + N + 1}{4(\nu + N_B)(\nu + N_R)}})$$

The cumulative normal distribution can be approximated by the logit function with rescaling $\phi(x) \approx \sigma(\sqrt{\frac{8}{\pi}}x)$ (ref Bishop figure 4.9), and $N_B - N_R = 2N(p_B - .5)$, yielding the sigmoidal form described above with

$$\kappa = 2N\sqrt{\frac{2(2\nu + N + 1)}{\pi(\nu + p_B N)(\nu + N(1 - p_B))}}$$

Here, parameter κ is dependent on p_B. The slope is approximated by taking the value around p_B =0.5, yielding:

$$\kappa = \frac{2N}{\nu + N/2}\sqrt{\frac{2}{\pi}(2\nu + N + 1)} \approx \frac{4N}{\sqrt{\pi(\nu + \frac{N}{2})}}$$

The slope scales as $\sqrt{N}$. In other words, the dependence of the optimal response on mean evidence increases with sample size in a sublinear fashion. The bias β is null, i.e. there an equal proportion of blue and red passenger should always give a center response.

Optimal inference with fixed symmetric probabilities

Here we assume that the generative process is taken as coming from a fixed probability μ or $1 - \mu$, with equal probability (so discrete priors instead of full distribution for priors). In such case the probability that the sample was generated from a process with a majority of blue planes amounts to inferring whether the process was generated with mean probability μ, which leads to :

$$\frac{c(B)}{c(R)} = (\frac{\mu}{1 - \mu})^{N_B - N_R}$$

This turns to:

$$c(B) = \sigma((N_B - N_R)\ln(\frac{\mu}{1-\mu}))$$

We recover the sigmoid shape with $\kappa = 2\ln(\frac{\mu}{1-\mu})N$, i.e. **the slope scales linearly with N**. It coincides in fact with the **difference model**. In other words, the difference model corresponds to the inference model when the uncertainty about the latent proportion is reduced to two possible (and symmetric) values.

- **Analytical approximation for Experiment 2**

We first derive approximations for the bottom-up messages $m_t(b)$ (in fact for the ratio between the message corresponding to both contexts), then for the top-down messages $M_t(b)$, and finally for the response itself.

Approximations for messages in the optimal model

$$\frac{m_t(1)}{m_t(0)} = \frac{\int_0^1 \mu^{\nu_1+N_{Bt}-1}(1-\mu)^{\nu_2+N_{Rt}-1}d\mu}{\int_0^1 \mu^{\nu_2+N_{Bt}-1}(1-\mu)^{\nu_1+N_{Rt}-1}d\mu} = \frac{\Gamma(\nu_2+N_{Bt})\Gamma(\nu_1+N_{Rt})}{\Gamma(\nu_1+N_{Bt})\Gamma(\nu_2+N_{Rt})} = \prod_{i=0}^{\nu_1-\nu_2-1} \frac{\nu_2+N_{Rt}+i}{\nu_2+N_{Bt}+i}$$

Using the Stirling's approximation for factorials and taking the logarithm

$$\log\frac{m_t(1)}{m_t(0)} \approx (\nu_2+N_{Bt})\log(\nu_2+N_{Bt}) + (\nu_1+N_{Rt})\log(\nu_1+N_{Rt}) - (\nu_1+N_{Bt})\log(\nu_1+N_{Bt}) - (\nu_2+N_{Rt})\log(\nu_2+N_{Rt})$$

As a zero-order approximation, by assuming $N_{Bt}, N_{Rt} \ll \nu_1, \nu_2$, we obtain

$$\log\frac{m_t(1)}{m_t(0)} \approx (N_{Bt} - N_{Rt})\log({}^{\nu_2}/_{\nu_1})$$

At first-order approximation $\log(\nu_i + N_{Bt}) \approx \log(\nu_i) + \frac{N_{Bt}}{\nu_i}$

$$\log\frac{m_t(1)}{m_t(0)} \approx (N_{Bt} - N_{Rt})\left[\log({}^{\nu_2}/_{\nu_1}) + N_t({}^{1}/_{\nu_2} - {}^{1}/_{\nu_1})\right]$$

Then the belief about block tendency can be written as:

$$\log\frac{M_t(1)}{M_t(0)} = \log\frac{p(b=1)}{p(b=0)} + \sum_{u=1}^{T-1}\log\frac{m_u(1)}{m_u(0)}$$

Such belief can be update at every trial using the classical equation for Sequential Probability Ratio Test:

$$\log\frac{M_{t+1}(1)}{M_{t+1}(0)} = \log\frac{M_t(1)}{M_t(0)} + \log\frac{m_t(1)}{m_t(0)}$$

Since priors for the two categories are equal, the first term vanishes. We have:

$$M_t(1) = \sigma(\sum_{u=1}^{T-1}\log\frac{m_u(1)}{m_u(0)})$$

In the zero-order approximation, we see that the belief about tendency simply depends on the overall difference between red dots and blue dots summed over previous trials:

$$M_t(1) \approx \sigma(\log({}^{\nu_2}/_{\nu_1}) \sum_{u=1}^{T-1} (N_{Bu} - N_{Ru}))$$

This is the **difference model** described in equation (12), with $\omega = \log({}^{\nu_2}/_{\nu_1})$but **without the normalization** by the number of previous trials (T-1) (i.e. the *total difference*, not the average difference). When the belief term remains in the linear portion of the logistic sigmoid (i.e. when the belief does not saturate towards either prior), the approximation becomes:

$$M_t(1) \approx \frac{1}{2} + \frac{1}{4}\log({}^{\nu_2}/_{\nu_1}) \sum_{u=1}^{T-1} (N_{Bu} - N_{Ru})$$

Approximation for model response

$$c(B|D) = \int_{\mu>0.5} \sum_{b\in\{0,1\}} M_t(b) Beta(\mu;\ \nu_{1b} + N_{BT}, \nu_{2b} + N_{RT})\, d\mu$$

where $\nu_{10} = \nu_2, \nu_{20} = \nu_2, \nu_{11} = \nu_1, \nu_{21} = \nu_2$. Using the Gaussian approximation for the Beta distribution

$$c(B|D) \approx \int_{\mu>0.5} \sum_{b\in\{0,1\}} M_t(b) N\left(\mu;\ \frac{\nu_{1b} + N_{BT}}{S + N_T}, \frac{(\nu_{1b} + N_{BT})(\nu_{2b} + N_{RT})}{(S + N_T)^2 (S + N_T + 1)}\right) d\mu$$

with $S = \nu_1 + \nu_2$.The weighted sum of gaussian can be turned into a single gaussian:

$$c(B|D) \approx \int_{\mu>0.5} N\left(\mu;\ \frac{N_{BT} + \sum_b M_t(b)\nu_{1b}}{S + N_T}, \frac{\sum_b M_t(b)\,(\nu_{1b} + N_{BT})(\nu_{2b} + N_{RT})}{(S + N_T)^2 (S + N_T + 1)}\right) d\mu$$

Using $\int_{x>a} N(x; m, v) dx = \ \phi(\frac{m-a}{\sqrt{v}})$, we get:

$$c(B|D) \approx \phi(0.5(N_{BT} - N_{RT} + 2\sum_b M_t(b)\nu_{1b} - S)\sqrt{\frac{S + N_T + 1}{\sum_b M_t(b)\,(\nu_{1b} + N_{BT})(\nu_{2b} + N_{RT})}})$$

The confidence takes the generic sigmoidal form described above where the **bias β depends on the belief about block tendency and is inversely proportional to the sample size of current trial.**

$$\beta = \frac{1}{2N_T}(M_t(0)\nu_2 + M_t(1)\nu_1 - S) = \frac{\Delta\nu}{2N_T}(2M_t(1) - 1)$$

With $\Delta\nu = \nu_1 - \nu_2$.

In the optimal model, when the belief does not saturate (see equation xx), the bias can be approximated by:

$$\beta \approx \frac{\Delta\nu}{2N_T}\left(2(\frac{1}{2} + \frac{1}{4}\log({}^{\nu_2}/_{\nu_1}) \sum_{u=1}^{T-1} (N_{Bu} - N_{Ru})) - 1\right)$$

i.e. $\beta \approx \frac{\Delta\nu}{4N_T}\log({}^{\nu_2}/_{\nu_1}) \sum_{u=1}^{T-1} (N_{Bu} - N_{Ru})$

The overall weight of previous trials scales (at 0th order) with $\Delta\nu \log({}^{\nu_2}/_{\nu_1})$. This shows that **if subjects use a prior that is more symmetric than the true one (**ν_2 closer to ν_1), **then previous trials will have a lower impact on decisions than the optimal model, as observed experimentally** (Figure 6-7).

The slope κ is:

$$\kappa = N_T \sqrt{\frac{8(S + N_T + 1)}{\pi(\nu_1\nu_2 + N_{BT}N_{RT} + M_t(1)(\nu_1 N_{RT} + \nu_2 N_{BT}) + M_t(0)(\nu_1 N_{BT} + \nu_2 N_{RT}))}}$$

$$\kappa = N_T \sqrt{\frac{8(S + N_T + 1)}{\pi((\nu_1 + N_{RT})(\nu_2 + N_{BT}) + M_t(1)\Delta\nu(N_{RT} - N_{BT}))}}$$

We can remove the dependence about block tendency by approximating $M_t(1) = 0.5$:

$$\kappa \approx 2N_T \sqrt{\frac{2(S + N_T + 1)}{\pi(\nu_1\nu_2 + N_{RT}N_{BT} + 0.5SN_T)}}$$

Supplementary figures

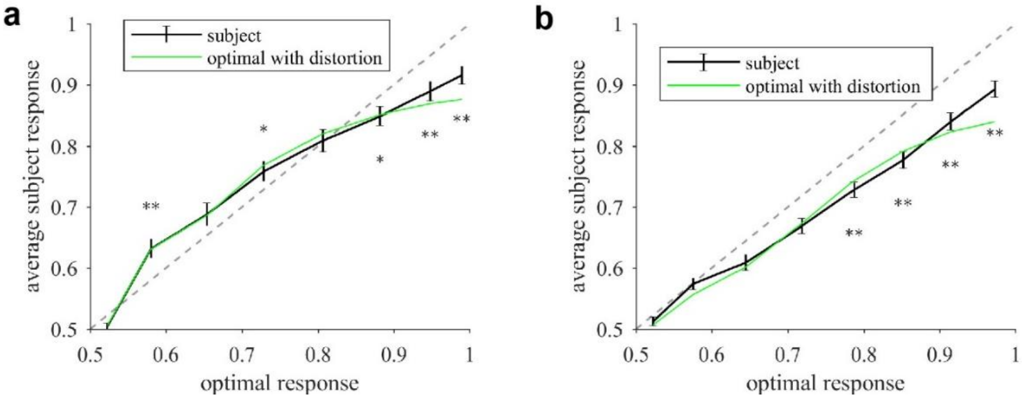

Supplementary Figure 1 Calibration of confidence judgments. Human confidence judgments (black, mean $\pm$ SEM) as a function of the response of the ideal observer (optimal response). Responses are grouped in approximately equally filled bins for Experiment 1 **(a)** and Experiment 2 **(b)**. Green curves represent the average responses of the optimal model including a flexible mapping from estimate to response ("distortion") that is fitted on participant responses individually. In Experiment 1, the correlation of decision confidence with the optimal response (i.e. the actual probability that the current trial's airplane passenger majority is blue) is $\rho = 0.81$ (Pearson coefficient, $p = 1.27 \cdot 10^{-45}$, see panel a). Similarly, in Experiment 2, the correlation of decision confidence with the optimal response is $\rho = 0.90$ (Pearson coefficient, $p = 6.8 \cdot 10^{-72}$, panel b). In both experiments, decision confidence shows systematic deviations from calibrated responses in that the participants are under-confident for difficult decisions of low expected accuracy; moreover, participants were overconfident for easy decisions in Experiment 1. Significant signed differences of the group median against calibrated responses are computed from a signed rank test and indicated for each bin ($* : 0.01 < p \leq 0.05$ and $** : p \leq 0.01$).

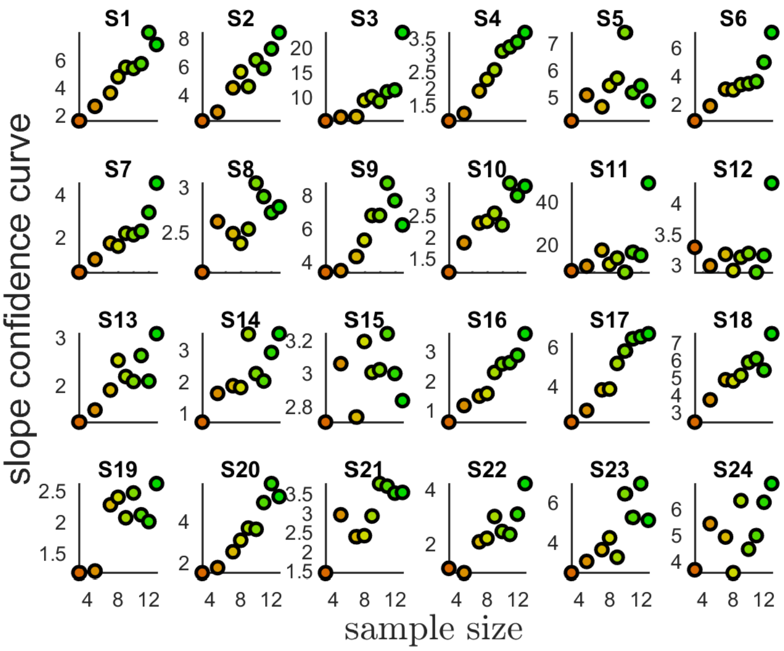

Supplementary Figure 2. Most subjects display sample size sensitivity. Slope of the confidence curve as a function of sample size, separately for each participant. 21 out of 24 participants showed significant correlation of the slope of the confidence curve with sample size (Pearson correlation at p<0.05, permutation test, uncorrected).

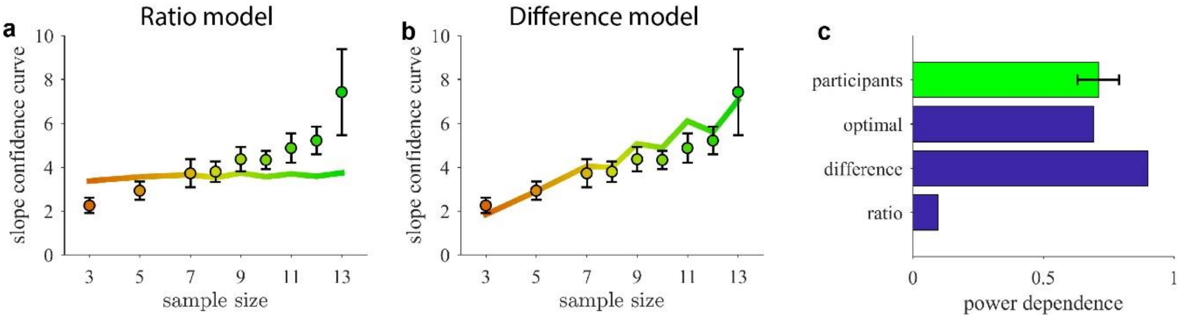

Supplementary Figure 3. Heuristics qualitative predictions are invalidated by participants behavior. a). Slope of the psychometric curve as a function of sample size in participants (error bars) and according to the ratio model (see section '*Experiment 1: predictions from heuristics models*' in Supplementary Methods). As predicted, the ratio model is completely insensitive to sample size. **b)** Slope of the psychometric cruve as a function of sample size in participants and according to the difference model. Slopes in the difference model qualitatively matched that of participants. The goodness of fit of the difference model is roughly similar to that of the optimal model (figure 2b): underestimation of slope is larger in the optimal model for small sample sizes, but the discrepancy with participant data is larger for sample size 9-11 in the difference model. Remember that this comparison is made on data averaged over participants. This motivated us to look for a single metric that can be estimated on each participant and allow more fine-grained comparison between the models. **c)** comparison of the power law coefficient (*b*) between participants and the different models (optimal, difference and ratio; see section '*Experiment 1: Predictions of heuristics models*'). We found that participants' slope depended on sample size in a sublinear way ($b<1$), exactly was for the optimal model (average power for the participants: 0.71; for the optimal model: 0.69). By contrast the power was close to 1 for the difference model and close to 0 for the ratio model (the values were not exactly 1 and 0 because of the distortions caused by the fitted mapping from estimate to response). Participants slope power was statistically different from that of the ratio model (paired t-test, $p<10^{-6}$) and that of the difference model ($p<0.02$), but not from that of the optimal model ($p>0.8$).

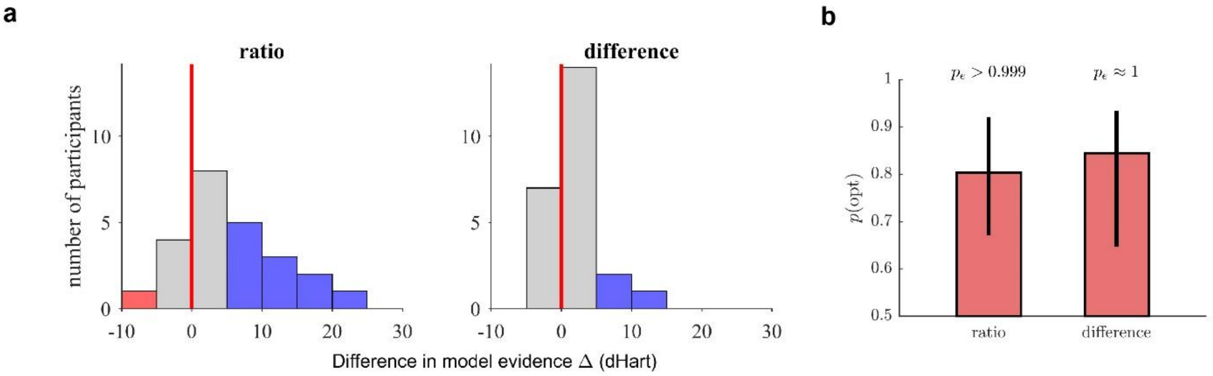

Supplementary Figure 4. Sample size is crucial to predict confidence judgments in Experiment 1. a) Histogram of difference of model evidence Δ (difference between cross-validated log-likelihood) between optimal model and heuristic models for all participants. Differences in model evidence, taken as the difference between the CVLL of two models for the same dataset, are reported on a log-scale in decibans (also decihartleys, abbreviated dHart) that may be used to interpret the significance of the results of individual participants. Left panel shows difference between the optimal model w.r.t. ratio model; right panel shows the difference between the optimal model w.r.t to the difference model. Positive values indicate that the optimal model better predicted participant response than the heuristic model. According to standard conventions, we consider a value of $5 > \Delta$ barely worth mentioning, $10 > \Delta \geq 5$ substantial, $15 > \Delta \geq 10$ strong, $20 > \Delta \geq 15$ very strong and $\Delta \geq 20$ decisive. **b)** Binomial probability of the optimal model to account for the data of a randomly chosen participant (error bars are 95 %-CI, Methods). Pairwise comparisons to the models (ratio, diff) show that probabilistic information integration yields better predictions on the group level. Additionally, the exceedance probability p_e is used to quantify how much more likely the optimal model is.

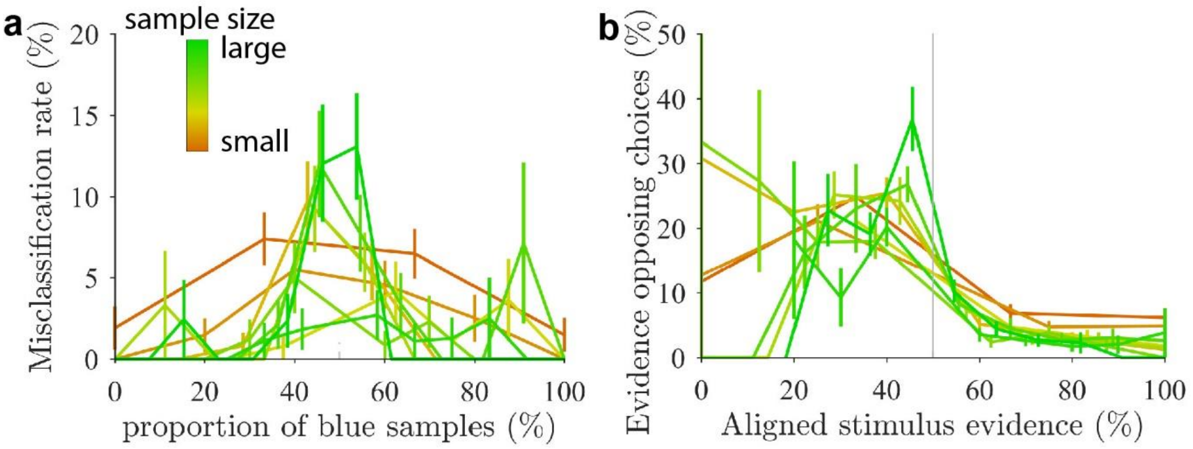

Supplementary Figure 5. Evidence-opposing choices are consistent with low sensory noise and influence of overall context. A. Misclassification rate as a function of blue samples and sample size in Experiment 1. Misclassified responses are rare and occur mostly for proportions of blue samples close to 50%. They tend to occur more frequently for larger sample sizes (color code). B. Evidence-opposing choice rate as a function of aligned proportion in Experiment 2. Evidence-opposing choices tend to occur when aligned stimulus evidence is lower than 50%, i.e when the more frequent colour in the current sample is at odds with the dominant color in the block, showing the influence of context in participant decisions.

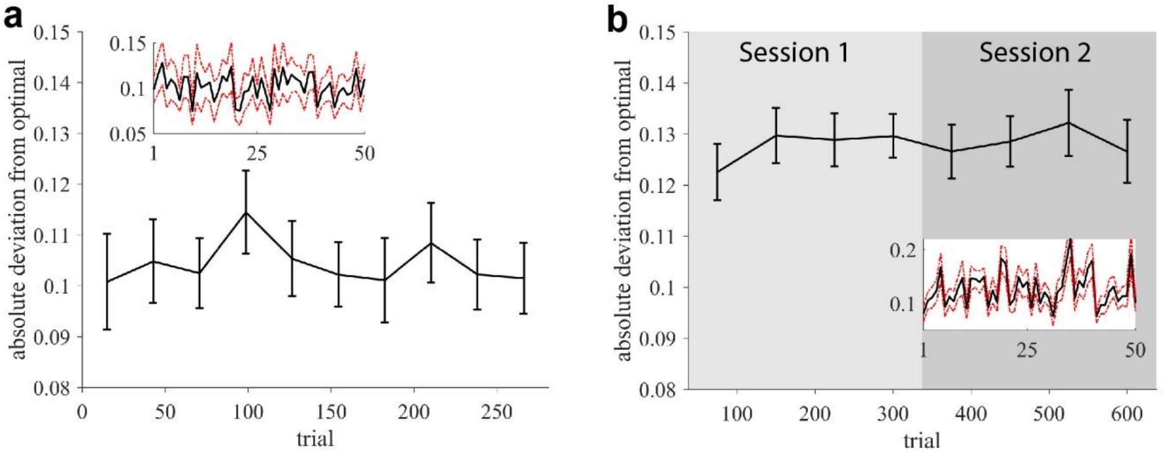

Supplementary Figure 6. Subjects performance was stable over the sessions in both experiments. Average absolute deviation of participant responses from optimal response as a function of trial index (mean and SEM across participants), for Experiment 1 (a) and Experiment 2 (performed in two different sessions) (**b**). Insets represent values for the first 50 trials.

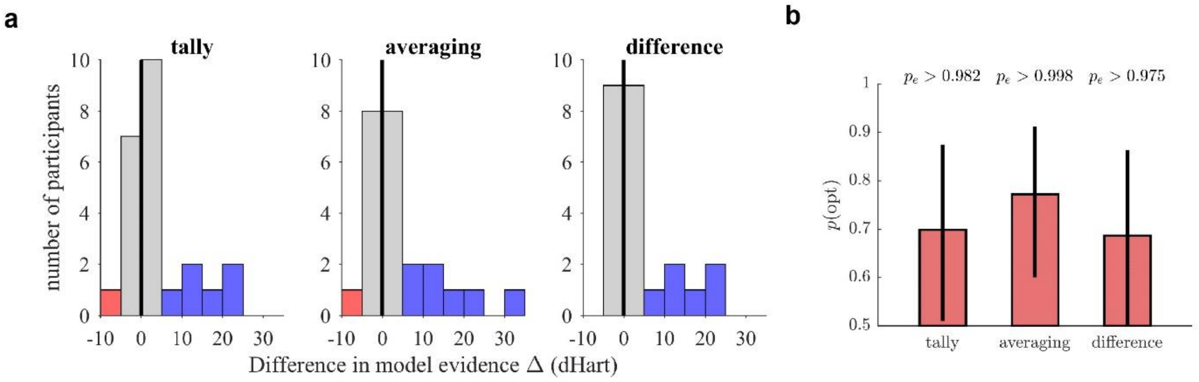

Supplementary Figure 7. Probabilistic estimation of the block tendency outperforms all heuristic approaches in Experiment 2. Instead of resorting to probabilistic inference, our participants might have used a heuristic way of estimating the block tendency. To determine whether that was the case, we performed a model comparison approach. The output of each model is passed through a generalization of the sigmoidal response mapping (Equations (13-14)) to impose fewest restriction on how participants might integrate the estimate of the block tendency with information from the current sample. **a.** Histogram of difference of model evidence Δ (difference between cross-validated log-likelihood) across all participants. Left panel shows difference between the optimal model w.r.t. averaging model, middle panel shows the difference w.r.t. the difference model, and right panel shows the difference w.r.t to the tally model. Positive values indicate that the optimal model better predicted participant response than the heuristic model. **b.** Binomial probability of the ideal observer model to account for the data of a randomly chosen participant (error bars are 95 %-CI, Methods). Pairwise comparisons to the models (tly, avg, diff) show that probabilistic information integration yields better predictions on the group level. Additionally, the exceedance probability p_e is used to quantify how much more likely the optimal model is.

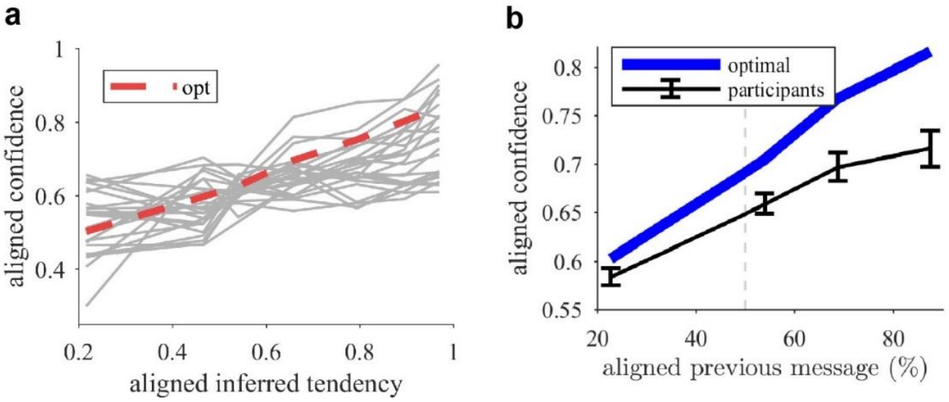

Supplementary Figure 8. Confirmatory analyses for hierarchical inference. a) Aligned confidence as a function of aligned inferred tendency *M(b)* for individual subjects (gray lines). Aligned confidence was correlated with aligned inferred tendency in 17 out of 24 participants (Pearson correlation, $p<0.05$ uncorrected). Response from optimal model is shown in red dashed line. **b)** Aligned confidence increases with aligned previous message $m_{r-1}(b)$ both in the optimal model (blue line) and in individual participants (lines and errors bars show mean and s.e.m. across participants respectively). Such analysis only looks at the influence of the previous stimulus, while the analysis of figure 5b represents the dependence of aligned confidence on aligned inferred tendency $M_t(b)$, which recapitulates the information provided by all previous stimuli in the block.

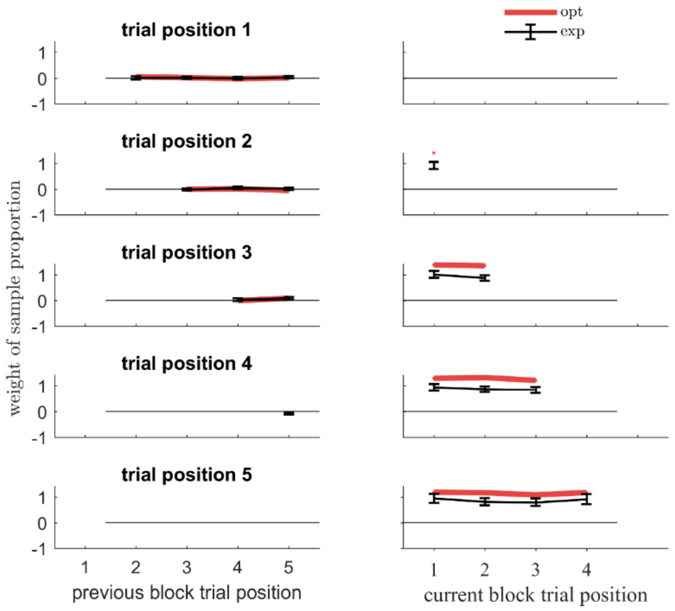

Supplementary Figure 9. Participant responses were not contaminated by previous block stimuli. Logistic regression analyses evaluated the impact from mean evidence of the four previous trials onto the responses depending on trial position within the block (from trial position 1, top panel; to trial position 5, bottom panel), for the optimal model (red curve) and participants (black curve: mean; error bars: SEM). In the optimal model, previous trials only impacted current response insofar as they pertained to the same block (right panels), and not if they corresponded to the previous block (left panels), as the belief about block tendency *M(b)* only accumulates messages from current block. This was also the case for participants, whose response was unaffected by stimuli corresponding to the previous block: in all cases the p-value associated the logistic regression weight (Wald t-test, uncorrected) was not larger than 0.1. This shows that participants made full use of the instruction that the context was drawn independently for each block, as their responses were not contaminated by previous block stimuli.

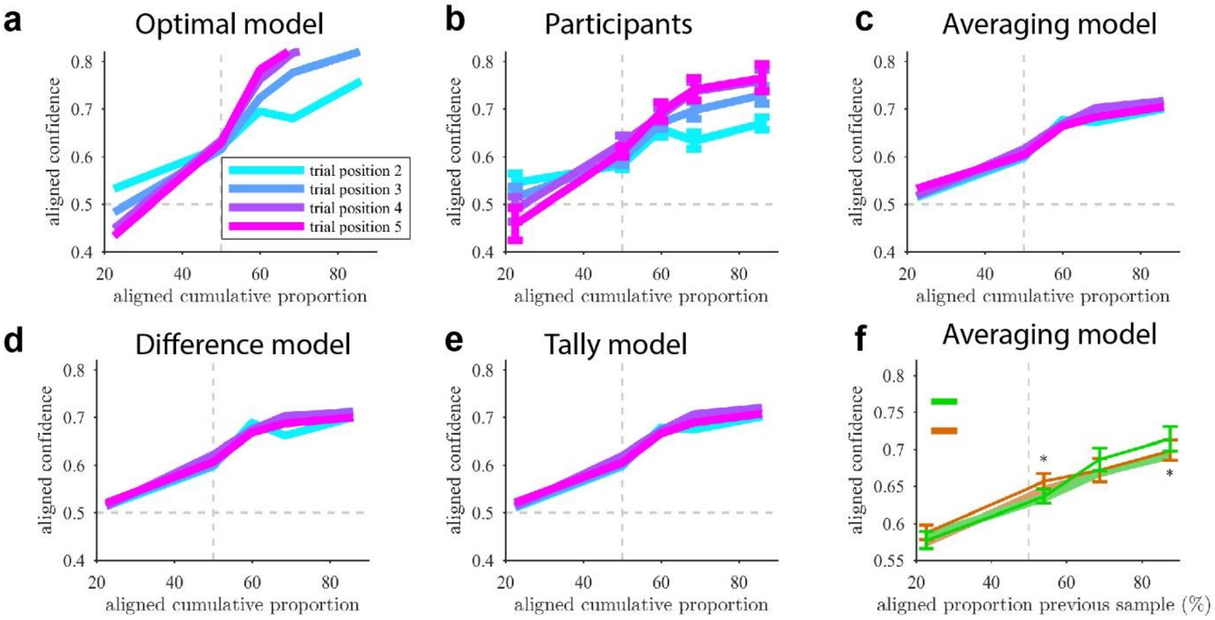

Supplementary Figure 10. Heuristics models fail to explain qualitative features of participant behavior in Experiment 2. Aligned confidence as a function of aligned cumulative proportion, i.e. the overall proportion of passengers corresponding to the real block tendency in previous trials in the block, and trial position (from trial position 2 in light blue to trial position 5 in magenta) **(a-e).** In the optimal model (a), a higher aligned cumulative proportion will correspond in general to a larger prior for the corresponding category and thus a larger aligned confidence. Moreover, the effects accumulate over trials in a block so that the sensitivity to accumulated proportion increases over trials trial in the block (see section 'Analytical approximation' for further insights). This is shown by an increasingly larger slope for larger trial number. Participants displays the same property (b). By contrast, in all heuristic models (c-e), the slope of dependence of aligned confidence on tally did not depend on trial position. **f)** By construction, the averaging model does not take into account sample size of previous stimuli to estimate the current context, and thus fails to display sensitivity to previous trial sample size as participants (Figure 6c).

Supplementary Table 1: Estimate of the compliance with the hierarchical task of the least engaged participants. Participants are ordered from left to right according to decreasing p-values.

	1	2	3	4	5	6
Δa	0.0037	0.0201	0.0258	0.0301	0.0224	0.0556
p-value	0.4372	0.2154	0.1415	0.0969	0.0894	0.0190

SUPPLEMENTARY REFERENCE

[1] P. Kramer, M. G. Di Bono, and M. Zorzi, "Numerosity estimation in visual stimuli in the absence of luminance-based cues," *PLoS One*, 2011.
